# Supplementary material for: Association between oral microbiome and seven types of cancers in East Asian population: a two-sample Mendelian randomization analysis
Source: Front Mol Biosci. 2023 Nov 21;10:1327893. doi: 10.3389/fmolb.2023.1327893 (PMC10702768; doi:10.3389/fmolb.2023.1327893)
Supplement: Supplementary file 1 [file DataSheet1.ZIP › Supplementary Materials/MR plots for tongue/tongue═╝/Lung cancer/pheno.3217_to_lung cancer_forest.pdf]

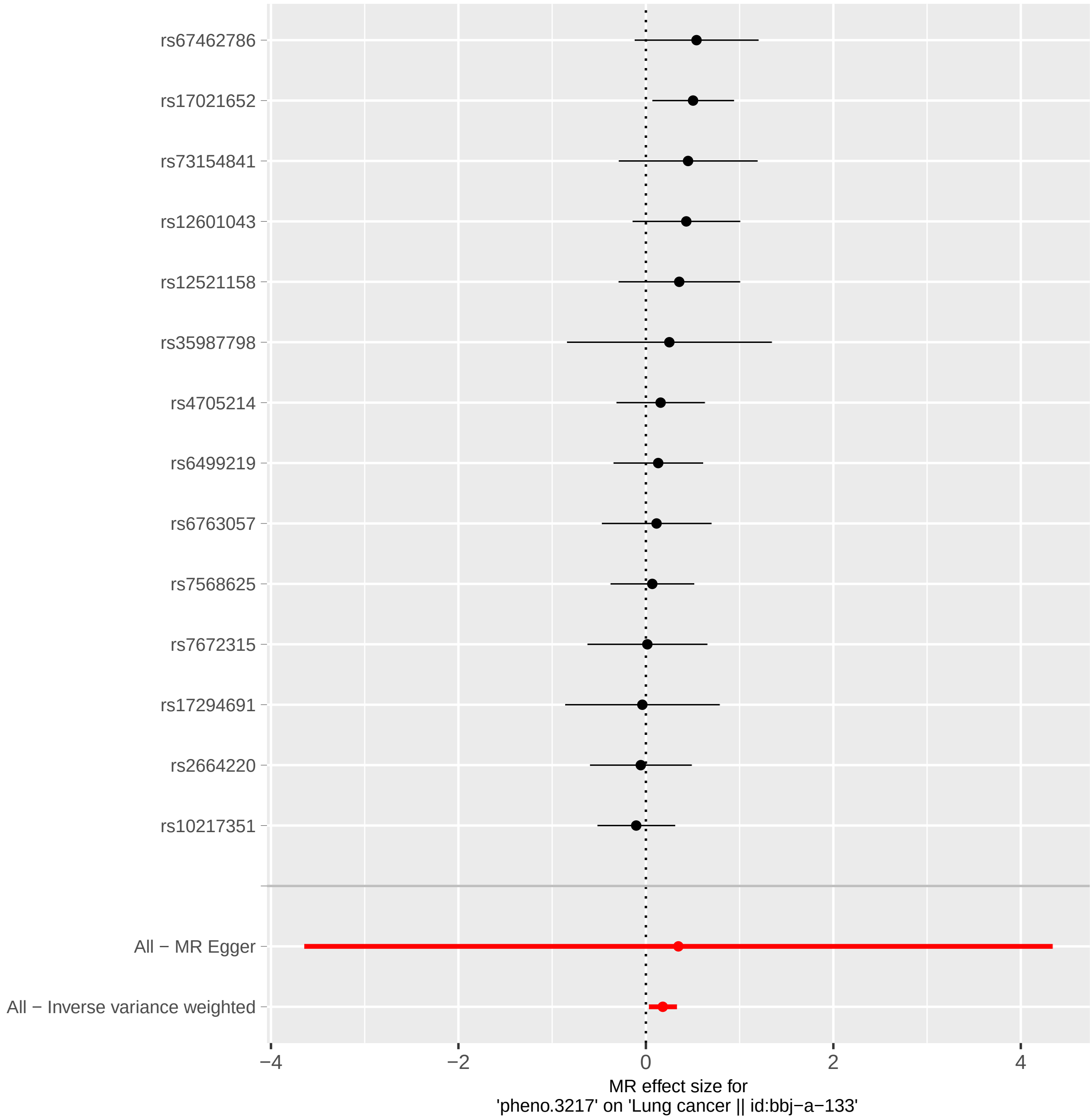
rs67462786
rs17021652
rs73154841
rs12601043
rs12521158
rs35987798
rs4705214
rs6499219
rs6763057
rs7568625
rs7672315
rs17294691
rs2664220
rs10217351
All − MR Egger
All − Inverse variance weighted
−4
−2
0
2
4
MR effect size for
'pheno.3217' on 'Lung cancer || id:bbj−a−133'
